# Early Estimates of Bivalent mRNA Vaccine Effectiveness in Preventing COVID-19–Associated Emergency Department or Urgent Care Encounters and Hospitalizations Among Immunocompetent Adults — VISION Network, Nine States, September–November 2022

**DOI:** 10.15585/mmwr.mm7153a1

**Published:** 2023-03-17

**Authors:** Mark W. Tenforde, Zachary A. Weber, Karthik Natarajan, Nicola P. Klein, Anupam B. Kharbanda, Edward Stenehjem, Peter J. Embi, Sarah E. Reese, Allison L. Naleway, Shaun J. Grannis, Malini B. DeSilva, Toan C. Ong, Manjusha Gaglani, Jungmi Han, Monica Dickerson, Bruce Fireman, Kristin Dascomb, Stephanie A. Irving, Gabriela Vazquez-Benitez, Suchitra Rao, Deepika Konatham, Palak Patel, Kristin E. Schrader, Ned Lewis, Nancy Grisel, Charlene McEvoy, Kempapura Murthy, Eric P. Griggs, Elizabeth A. K. Rowley, Ousseny Zerbo, Julie Arndorfer, Margaret M. Dunne, Kristin Goddard, Caitlin Ray, Yan Zhuang, Julius Timbol, Morgan Najdowski, Duck-Hye Yang, John Hansen, Sarah W. Ball, Ruth Link-Gelles

**Affiliations:** ^1^Influenza Division, National Center for Immunization and Respiratory Diseases, CDC; ^2^Westat Inc., Rockville, Maryland; ^3^Department of Biomedical Informatics, Columbia University Irving Medical Center, New York, New York; ^4^New York Presbyterian Hospital, New York, New York; ^5^Kaiser Permanente Vaccine Study Center, Kaiser Permanente Northern California Division of Research, Oakland, California; ^6^Children's Minnesota, Minneapolis, Minnesota; ^7^Division of Infectious Diseases and Clinical Epidemiology, Intermountain Healthcare, Salt Lake City, Utah; ^8^Vanderbilt University Medical Center, Nashville, Tennessee; ^9^Center for Biomedical Informatics, Regenstrief Institute, Indianapolis, Indiana; ^10^Kaiser Permanente Center for Health Research, Portland, Oregon; ^11^School of Medicine, Indiana University, Indianapolis, Indiana; ^12^HealthPartners Institute, Minneapolis, Minnesota; ^13^School of Medicine, University of Colorado Anschutz Medical Campus, Aurora, Colorado; ^14^Department of Pediatrics, Section of Pediatric Infectious Diseases, Baylor Scott & White Health, Temple, Texas; ^15^Department of Medical Education, Texas A&M University College of Medicine, Temple, Texas; ^16^Department of Research Analytics and Development, Baylor Scott & White Research Institute, Baylor Scott & White Health, Temple Texas; ^17^National Center for Immunization and Respiratory Diseases, CDC.

During June–October 2022, the SARS-CoV-2 Omicron BA.5 sublineage accounted for most of the sequenced viral genomes in the United States, with further Omicron sublineage diversification through November 2022.* Bivalent mRNA vaccines contain an ancestral SARS-CoV-2 strain component plus an updated component of the Omicron BA.4/BA.5 sublineages. On September 1, 2022, a single bivalent booster dose was recommended for adults who had completed a primary vaccination series (with or without subsequent booster doses), with the last dose administered ≥2 months earlier (*1*). During September 13–November 18, the VISION Network evaluated vaccine effectiveness (VE) of a bivalent mRNA booster dose (after 2, 3, or 4 monovalent doses) compared with 1) no previous vaccination and 2) previous receipt of 2, 3, or 4 monovalent-only mRNA vaccine doses, among immunocompetent adults aged ≥18 years with an emergency department/urgent care (ED/UC) encounter or hospitalization for a COVID-19–like illness.^†^ VE of a bivalent booster dose (after 2, 3, or 4 monovalent doses) against COVID-19–associated ED/UC encounters was 56% compared with no vaccination, 32% compared with monovalent vaccination only with last dose 2–4 months earlier, and 50% compared with monovalent vaccination only with last dose ≥11 months earlier. VE of a bivalent booster dose (after 2, 3, or 4 monovalent doses) against COVID-19–associated hospitalizations was 59% compared with no vaccination, 42% compared with monovalent vaccination only with last dose 5–7 months earlier, and 48% compared with monovalent vaccination only with last dose ≥11 months earlier. Bivalent vaccines administered after 2, 3, or 4 monovalent doses were effective in preventing medically attended COVID-19 compared with no vaccination and provided additional protection compared with past monovalent vaccination only, with relative protection increasing with time since receipt of the last monovalent dose. All eligible persons should stay up to date with recommended COVID-19 vaccinations, including receiving a bivalent booster dose. Persons should also consider taking additional precautions to avoid respiratory illness this winter season, such as masking in public indoor spaces, especially in areas where COVID-19 community levels are high.

Monovalent COVID-19 mRNA vaccines were developed against the spike protein of the ancestral SARS-CoV-2 virus and were found to provide cross-reactive immune protection against Alpha and Delta SARS-CoV-2 variants (*2*). The SARS-CoV-2 Omicron variant emerged in November 2021 and diversified into sublineages. These Omicron sublineages were associated with decreased protection from vaccination with monovalent vaccine (*3*). A single booster dose of bivalent mRNA vaccine (Pfizer-BioNTech or Moderna) containing an updated BA.4/BA.5 component was recommended by CDC on September 1, 2022, (*1*) for adults who had completed a primary series with any Food and Drug Administration–approved or –authorized monovalent vaccine or who had previously received a monovalent booster dose ≥2 months earlier.^§^

The VISION Network^¶^ evaluated the effectiveness of a bivalent booster dose among immunocompetent adults during September 13–November 18, 2022, a period during which the Omicron BA.5 sublineage predominated and additional Omicron sublineages emerged. Seven health systems in nine states contributed data for this analysis. VISION methods have been described (*3*). Briefly, ED/UC encounters and hospitalizations associated with a COVID-19–like illness among adults who received a SARS-CoV-2 molecular test result during the 14 days before through 72 hours after the encounter were included.** Patients were classified as unvaccinated (zero doses received), vaccinated with 2, 3, or 4 doses of a monovalent-only mRNA vaccine, or vaccinated with 2, 3, or 4 monovalent doses plus a bivalent booster dose ≥60 days after receipt of their last monovalent dose. Encounters were excluded if 1) the patient likely had an immunocompromising condition (*4*); 2) only one mRNA monovalent vaccine dose was received, a second monovalent vaccine dose was received <14 days before the encounter date, or a third or fourth monovalent vaccine dose or a bivalent booster dose was received <7 days before the encounter date; 3) any dose of a non-mRNA vaccine (e.g., Janssen [Johnson & Johnson]) was received; or 4) a vaccine dose was received before being recommended by CDC.^††^ VE was estimated using a test-negative case-control design, comparing the odds of having received versus having not received a bivalent booster dose among case-patients (those who received a positive SARS-CoV-2 test result) and control patients (those who received a negative SARS-CoV-2 test result). 

Odds ratios and 95% CIs were calculated using multivariable logistic regression, adjusting for age, race and ethnicity, sex, calendar day (days since January 1, 2021), geographic region, and local SARS-CoV-2 circulation (percentage of SARS-CoV-2–positive results from testing within the counties surrounding the facility on the date of the encounter). Age, calendar day, and local circulation were modeled as natural cubic splines. A single, combined model was fit for each outcome (ED/UC encounters and hospitalizations) with those who had received a bivalent booster dose (after 2, 3, or 4 monovalent doses) as the referent group with the following vaccination groups: those who had received no vaccine doses (unvaccinated) (i.e., absolute VE) and those who had received 2, 3, or 4 monovalent doses but not a bivalent booster dose (i.e., relative VE). Varying time intervals between the last dose and the index date (2–4, 5–7, 8–10, or ≥11 months)^§§^ were used to calculate relative VE. Analyses were conducted using R (version 4.2.2; R Foundation). This study was conducted consistent with applicable federal law and CDC policy and was reviewed and approved by Institutional Review Boards at participating sites or under reliance agreement with the Institutional Review Board of Westat, Inc.^¶¶^

Among 78,170 ED/UC encounters with COVID-19–like illness that met inclusion criteria, 8,986 (12%) case-patients and 69,184 (89%) control patients were identified (Table 1). Overall, 24,130 (31%) were unvaccinated. Among persons who had not received a bivalent dose, 18,724 (24%), 22,539 (29%), and 8,080 (10%) had received 2, 3, and 4 doses of monovalent mRNA vaccine, respectively. Among the 4,697 (6%) adults who had received a bivalent booster dose (median interval since receipt of bivalent booster dose = 25 days), 292 (6%) had received 2 monovalent doses, 2,115 (45%) had received 3 monovalent doses, and 2,290 (49%) had received 4 monovalent vaccine doses. Bivalent booster dose recipients were older (median age = 68 years) than were those who had not received a bivalent booster dose (median age = 55 years). VE of a bivalent booster dose (after 2, 3, or 4 monovalent doses) against ED/UC encounters for COVID-19–associated illness was 56% (95% CI = 50%–61%) compared with no vaccination, 32% (95% CI = 21%–42%) compared with receipt of last monovalent dose 2–4 months earlier, and 50% (95% CI = 44%–56%) compared with receipt of last monovalent dose ≥11 months earlier (Table 2).

**TABLE 1:** Characteristics of emergency department and urgent care encounters among immunocompetent adults aged ≥18 years with COVID-19–like illness,* by mRNA COVID-19 vaccination status and SARS-CoV-2 test result — nine states,**^†^** September–November 2022

Characteristic	Overall, no. (column %)	SARS-CoV-2 test result status, no. (row %)		SMD^¶^	mRNA COVID-19 vaccination status,^§^ no. (row %)						SMD^¶^
		Case-patients (positive)	Control patients (negative)		Unvaccinated	Received 2, 3, or 4 MV doses only, interval since last dose (mos)				Received BV booster dose ≥7 days earlier	
						2–4	5–7	8–10	≥11		
**All ED/UC encounters**	**78,170 (100.0)**	**8,986 (11.5)**	**69,184 (88.5)**	**—**	**24,130 (30.9)**	**5,571 (7.1)**	**6,623 (8.5)**	**14,050 (18.0)**	**23,099 (29.5)**	**4,697 (6.0)**	**—**
**Site**											
Baylor Scott & White Health	**13,516 (17.3)**	1,390 (10.3)	12,126 (89.7)	0.37	7,014 (51.9)	288 (2.1)	374 (2.8)	1,244 (9.2)	4,513 (33.4)	83 (0.6)	3.81
Columbia University	**3,243 (4.1)**	253 (7.8)	2,990 (92.2)		1,421 (43.8)	110 (3.4)	209 (6.4)	508 (15.7)	941 (29.0)	54 (1.7)	
HealthPartners	**14,214 (18.2)**	1,637 (11.5)	12,577 (88.5)		3,523 (24.8)	1,236 (8.7)	1,296 (9.1)	3,006 (21.1)	3,683 (25.9)	1,470 (10.3)	
Intermountain Healthcare	**15,977 (20.4)**	2,723 (17.0)	13,254 (83.0)		5,278 (33.0)	827 (5.2)	921 (5.8)	2,763 (17.3)	5,160 (32.3)	1,028 (6.4)	
KPNC	**19,484 (24.9)**	1,326 (6.8)	18,158 (93.2)		2,431 (12.5)	2,350 (12.1)	3,052 (15.7)	4,787 (24.6)	5,339 (27.4)	1,525 (7.8)	
KPCHR	**5,840 (7.5)**	736 (12.6)	5,104 (87.4)		1,405 (24.1)	617 (10.6)	602 (10.3)	1,190 (20.4)	1,611 (27.6)	415 (7.1)	
University of Colorado	**5,896 (7.5)**	921 (15.6)	4,975 (84.4)		3,058 (51.9)	143 (2.4)	169 (2.9)	552 (9.4)	1,852 (31.4)	122 (2.1)	
**Age group, yrs**											
18–49	**39,148 (50.1)**	4,031 (10.3)	35,117 (89.7)	0.14	16,465 (42.1)	858 (2.2)	1,645 (4.2)	7,118 (18.2)	11,865 (30.3)	1,197 (3.1)	3.38
50–64	**14,666 (18.8)**	1,708 (11.6)	12,958 (88.4)		3,899 (26.6)	1,339 (9.1)	1,272 (8.7)	3,044 (20.8)	4,250 (29.0)	862 (5.9)	
65–74	**10,509 (13.4)**	1,304 (12.4)	9,205 (87.6)		1,896 (18.0)	1,332 (12.7)	1,407 (13.4)	1,706 (16.2)	3,040 (28.9)	1,128 (10.7)	
75–84	**8,812 (11.3)**	1,266 (14.4)	7,546 (85.6)		1,201 (13.6)	1,253 (14.2)	1,449 (16.4)	1,412 (16.0)	2,483 (28.2)	1,014 (11.5)	
≥85	**5,035 (6.4)**	677 (13.4)	4,358 (86.6)		669 (13.3)	789 (15.7)	850 (16.9)	770 (15.3)	1,461 (29.0)	496 (9.9)	
**Sex**											
Female	**48,262 (61.7)**	5,331 (11.0)	42,931 (89.0)	0.06	14,547 (30.1)	3,377 (7.0)	4,029 (8.3)	8,931 (18.5)	14,603 (30.3)	2,775 (5.7)	0.15
Male	**29,908 (38.3)**	3,655 (12.2)	26,253 (87.8)		9,583 (32.0)	2,194 (7.3)	2,594 (8.7)	5,119 (17.1)	8,496 (28.4)	1,922 (6.4)	
**Race and ethnicity**											
Black or African American, NH	**9,260 (11.8)**	823 (8.9)	8,437 (91.1)	0.17	3,837 (41.4)	515 (5.6)	694 (7.5)	1,420 (15.3)	2,547 (27.5)	247 (2.7)	1.13
Hispanic or Latino	**14,689 (18.8)**	1,345 (9.2)	13,344 (90.8)		5,118 (34.8)	843 (5.7)	1,081 (7.4)	2,743 (18.7)	4,456 (30.3)	448 (3.0)	
Other, NH**	**7,412 (9.5)**	841 (11.3)	6,571 (88.7)		1,746 (23.6)	657 (8.9)	780 (10.5)	1,732 (23.4)	2,013 (27.2)	484 (6.5)	
Unknown	**1,255 (1.6)**	154 (12.3)	1,101 (87.7)		547 (43.6)	46 (3.7)	73 (5.8)	239 (19.0)	317 (25.3)	33 (2.6)	
White, NH	**45,554 (58.3)**	5,823 (12.8)	39,731 (87.2)		12,882 (28.3)	3,510 (7.7)	3,995 (8.8)	7,916 (17.4)	13,766 (30.2)	3,485 (7.7)	
**Documented previous SARS-CoV-2 infection^††^**											
Yes	**15,726 (20.1)**	1,244 (7.9)	14,482 (92.1)	0.19	4,680 (29.8)	1,020 (6.5)	1,322 (8.4)	2,875 (18.3)	5,066 (32.2)	763 (4.9)	0.15
No	**62,444 (79.9)**	7,742 (12.4)	54,702 (87.6)		19,450 (31.1)	4,551 (7.3)	5,301 (8.5)	11,175 (17.9)	18,033 (28.9)	3,934 (6.3)	
**SARS-CoV-2 status**											
Positive test result (case-patient)	**8,986 (11.5)**	8,986 (100.0)	0 (—)	—	3,037 (33.8)	531 (5.9)	679 (7.6)	1,663 (18.5)	2,738 (30.5)	338 (3.8)	0.25
Negative test result (control patient)	**69,184 (88.5)**	0 (—)	69,184 (100.0)		21,093 (30.5)	5,040 (7.3)	5,944 (8.6)	12,387 (17.9)	20,361 (29.4)	4,359 (6.3)	
**No. of MV mRNA vaccine doses received**											
None	**24,130 (30.9)**	3,037 (12.6)	21,093 (87.4)	0.08	24,130 (100.0)	0 (—)	0 (—)	0 (—)	0 (—)	0 (—)	—
2	**19,016 (24.3)**	2,157 (11.3)	16,859 (88.7)		0 (—)	274 (1.4)	595 (3.1)	1,384 (7.3)	16,471 (86.6)	292 (1.5)	
3	**24,654 (31.5)**	2,740 (11.1)	21,914 (88.9)		0 (—)	997 (4.0)	2,248 (9.1)	12,666 (51.4)	6,628 (26.9)	2,115 (8.6)	
4	**10,370 (13.3)**	1,052 (10.1)	9,318 (89.9)		0 (—)	4,300 (41.5)	3,780 (36.5)	0 (—)	0 (—)	2,290 (22.1)	
**Most recent dose product manufacturer**											
Pfizer-BioNTech	**34,705 (44.4)**	3,825 (11.0)	30,880 (89.0)	0.07	0 (—)	3,533 (10.2)	4,231 (12.2)	8,327 (24.0)	15,018 (43.3)	3,596 (10.4)	—
Moderna	**19,335 (24.7)**	2,124 (11.0)	17,211 (89.0)		0 (—)	2,038 (10.5)	2,392 (12.4)	5,723 (29.6)	8,081 (41.8)	1,101 (5.7)	
None	**24,130 (30.9)**	3,037 (12.6)	21,093 (87.4)		24,130 (100.0)	0 (—)	0 (—)	0 (—)	0 (—)	0 (—)	
**Any chronic condition**											
Yes	**23,834 (30.5)**	2,307 (9.7)	21,527 (90.3)	0.12	6,778 (28.4)	2,068 (8.7)	2,370 (9.9)	4,011 (16.8)	7,195 (30.2)	1,412 (5.9)	0.45
No	**54,336 (69.5)**	6,679 (12.3)	47,657 (87.7)		17,352 (31.9)	3,503 (6.4)	4,253 (7.8)	10,039 (18.5)	15,904 (29.3)	3,285 (6.0)	
**≥1 chronic respiratory condition**											
Yes	**12,290 (15.7)**	1,057 (8.6)	11,233 (91.4)	0.13	3,604 (29.3)	987 (8.0)	1,148 (9.3)	2,039 (16.6)	3,800 (30.9)	712 (5.8)	0.2
No	**65,880 (84.3)**	7,929 (12.0)	57,951 (88.0)		20,526 (31.2)	4,584 (7.0)	5,475 (8.3)	12,011 (18.2)	19,299 (29.3)	3,985 (6.0)	
**≥1 chronic non-respiratory condition**											
Yes	**17,215 (22.0)**	1,832 (10.6)	15,383 (89.4)	0.05	4,867 (28.3)	1,562 (9.1)	1,715 (10.0)	2,822 (16.4)	5,290 (30.7)	959 (5.6)	0.39
No	**60,955 (78.0)**	7,154 (11.7)	53,801 (88.3)		19,263 (31.6)	4,009 (6.6)	4,908 (8.1)	11,228 (18.4)	17,809 (29.2)	3,738 (6.1)	

**Abbreviations:** BV = bivalent; ED/UC = emergency department/urgent care; KPNC = Kaiser Permanente Northern California; KPCHR = Kaiser Permanente Center for Health Research; MV = monovalent; NH = non-Hispanic; SMD = standardized mean or proportion difference.

* ED/UC encounters with a discharge code consistent with COVID-19–like illness were included. COVID-19–like illness diagnoses included acute respiratory illness, respiratory signs or symptoms, or febrile signs or symptoms using diagnosis codes from the *International Classification of Diseases, Tenth Revision*. Clinician-ordered molecular assays (e.g., real-time reverse transcription–polymerase chain reaction) for SARS-CoV-2 occurring ≤14 days before to <72 hours after the encounter date were included.

^†^ California (Sep 13–Nov 18, 2022), Colorado (Sep 13–Nov 7, 2022), Minnesota and Wisconsin (Sep 13–Nov 18, 2022), New York (Sep 13–Nov 18, 2022), Oregon and Washington (Sep 13–Nov 14, 2022), Texas (Sep 13–Nov 13, 2022), and Utah (Sep 13–Nov 18, 2022).

^§^ Vaccination was defined as having received the last monovalent or bivalent dose within the specified range of months or days before the ED/UC encounter date, which was the date of respiratory specimen collection associated with the most recent positive or negative SARS-CoV-2 test result before the encounter start date or the encounter start date if testing only occurred after the admission.

^¶^ An absolute SMD >0.20 indicates a nonnegligible difference in variable distributions between ED/UC encounters for vaccinated versus unvaccinated patients or for patients with positive SARS-CoV-2 test results versus patients with negative SARS-CoV-2 test results. For mRNA COVID-19 vaccination status, a single SMD was calculated by averaging the absolute SMDs obtained from pairwise comparisons of each vaccinated category versus unvaccinated. Specifically, it was calculated as the average of the absolute value of the SMDs for 1) vaccinated with only monovalent doses, ≥11 months earlier versus unvaccinated, 2) vaccinated with only monovalent doses, 8–10 months earlier versus unvaccinated, 3) vaccinated with only monovalent doses 5–7 months earlier versus unvaccinated, 4) vaccinated with only monovalent doses 2–4 months earlier versus unvaccinated, and 5) vaccinated with bivalent booster ≥7 days earlier versus unvaccinated.

** Other race includes Asian, Hawaiian or other Pacific Islander, American Indian or Alaska Native, other not listed, and multiple races. Because of small numbers, these categories were combined.

**^††^** Previous SARS-CoV-2 infection was defined as having a positive SARS-CoV-2 test result (molecular or antigen) documented in the electronic health record ≥15 days before the hospital admission date. This does not capture previous infections in which testing was not performed or testing was performed but not available in the electronic health record (e.g., at-home testing).

**TABLE 2:** Bivalent booster COVID-19 vaccine effectiveness* against laboratory confirmed COVID-19–associated emergency department and urgent care encounters and hospitalizations among immunocompetent adults aged 18 years — nine states,**^†^** September–November 2022

mRNA dosage pattern	Total	Negative SARS-CoV-2 test result, no. (%)	Positive SARS-CoV-2 test result, no. (%)	Median interval since last dose, days (IQR)	VE % (95% CI)
**ED/UC encounters**					
**Relative VE**					
Only MV doses, last dose 2–4 mos earlier	**5,571**	5,040 (91)	531 (10)	115 (91–134)	Ref
BV booster dose, ≥7 days earlier	**4,697**	4,359 (93)	338 (7)	25 (16–37)	32 (21–42)
Only MV doses, last dose 5–7 mos earlier	**6,623**	5,944 (90)	679 (10)	184 (166–210)	Ref
BV booster dose, ≥7 days earlier	**4,697**	4,359 (93)	338 (7)	25 (16–37)	43 (34–50)
Only MV doses, last dose 8–10 mos earlier	**14,050**	12,387 (88)	1,663 (12)	294 (273–311)	Ref
BV booster dose, ≥7 days earlier	**4,697**	4,359 (93)	338 (7)	25 (16–37)	54 (48–59)
Only MV doses, last dose ≥11 mos earlier	**23,099**	20,361 (88)	2,738 (12)	463 (366–543)	Ref
BV booster dose, ≥7 days earlier	**4,697**	4,359 (93)	338 (7)	25 (16–37)	50 (44–56)
**Absolute VE**					
Unvaccinated	**24,130**	21,093 (87)	3,037 (13)	NA	Ref
BV booster dose, ≥7 days earlier	**4,697**	4,359 (93)	338 (7)	25 (16–37)	56 (50–61)
**Hospitalizations**					
**Relative VE**					
Only MV doses, last dose 2–4 mos earlier	**—** ^§^	—	—	—	—
BV booster dose, ≥7 days earlier	**—**	—	—	—	—
Only MV doses, last dose 5–7 mos earlier	**1,780**	1,615 (91)	165 (9)	178 (164–201)	Ref
BV booster dose, ≥7 days earlier	**884**	828 (94)	56 (6)	23 (14–34)	42 (20–58)
Only MV doses, last dose 8–10 mos earlier	**2,636**	2,405 (91)	231 (9)	294 (273–313)	Ref
BV booster dose, ≥7 days earlier	**884**	828 (94)	56 (6)	23 (14–34)	44 (23–59)
Only MV doses, last dose ≥11 mos earlier	**4,549**	4,105 (90)	444 (10)	474 (362–556)	Ref
BV booster dose, ≥7 days earlier	**884**	828 (94)	56 (6)	23 (14–34)	48 (30–62)
**Absolute VE**					
Unvaccinated	**4,092**	3,658 (89)	434 (11)	NA	Ref
BV booster dose, ≥7 days earlier	**884**	828 (94)	56 (6)	23 (14–34)	59 (44–70)

**Abbreviations**: BV = bivalent; ED/UC = emergency department/urgent care; MV = monovalent; NA = not applicable; Ref = referent group; VE = vaccine effectiveness.

* VE was calculated as ([1 − odds ratio] x 100%), estimated using a test-negative case-control design, adjusted for age, sex, race and ethnicity, geographic region, calendar time (days since January 1, 2021), and local virus circulation (percentage of positive SARS-CoV-2 test results from testing within the counties surrounding the facility on the date of the encounter).

^†^ California (Sep 13, 2022–Nov 18, 2022), Colorado (Sep 13, 2022–Nov 7, 2022), Minnesota and Wisconsin (Sep 13, 2022–Nov 18, 2022), New York (Sep 13, 2022–Nov 18, 2022), Oregon and Washington (Sep 13, 2022–Nov 14, 2022), Texas (Sep 13, 2022–Nov 13, 2022), and Utah (Sep 13, 2022–Nov 18, 2022).

^§^ Dashes indicate that estimated VE had a CI width ≥50%. Estimates with CI widths ≥50% are not shown here because of imprecision. The associated data are also omitted.

Among 15,502 hospitalizations with COVID-19–like illness that met inclusion criteria, 1,452 (9%) case-patients and 14,050 (91%) control patients were identified (Table 3). Overall, 4,092 (26%) were unvaccinated. Among those who had not received a bivalent dose, 3,343 (22%), 4,704 (30%), and 2,479 (16%) had received 2, 3, and 4 doses of monovalent mRNA vaccine, respectively. Among the 884 (6%) adults who had received a bivalent booster dose (median interval since receipt of bivalent booster dose = 23 days), 58 (7%) had received 2 monovalent doses, 299 (34%) had received 3 monovalent doses, and 527 (60%) had received 4 monovalent doses. Bivalent booster dose recipients were similar in age to vaccinated adults who had not received a bivalent booster dose (median age = 76 and 73 years, respectively). VE of a bivalent booster dose (after 2, 3, or 4 monovalent doses) against hospitalization for COVID-19–associated illness was 59% (95% CI = 44%–70%) compared with no vaccination and 48% (95% CI = 30%–62%) compared with receipt of last monovalent doses, with last dose ≥11 months earlier (Table 2).

**TABLE 3:** Characteristics of hospitalizations among immunocompetent adults aged ≥18 years with COVID-19–like illness,* by mRNA COVID-19 vaccination status and SARS-CoV-2 test result — nine states,**^†^** September–November 2022

Characteristic	Overall, no. (col %)	SARS-CoV-2 test result status, no. (row %)		SMD^¶^	mRNA COVID-19 vaccination status.^§^ no. (row %)						SMD^¶^
		Case-patients (positive)	Control patients (negative)		Unvaccinated	Received 2, 3, or 4 MV doses only, interval since last dose (mos)				Received BV booster dose ≥7 days earlier	
						2–4	5–7	8–10	≥11		
**All hospitalizations**	**15,502 (100.0)**	**1,452 (9.4)**	**14,050 (90.6)**	**—**	**4,092 (26.4)**	**1,561 (10.1)**	**1,780 (11.5)**	**2,636 (17.0)**	**4,549 (29.3)**	**884 (5.7)**	**—**
**Site**											
Baylor Scott & White Health	**3,782 (24.4)**	331 (8.8)	3,451 (91.2)	0.2	1,545 (40.9)	117 (3.1)	136 (3.6)	433 (11.4)	1,516 (40.1)	35 (0.9)	3.92
Columbia University	**1,125 (7.2)**	128 (11.4)	997 (88.6)		432 (38.4)	69 (6.1)	109 (9.7)	200 (17.8)	292 (26.0)	23 (2.0)	
HealthPartners	**1,504 (9.7)**	165 (11.0)	1,339 (89.0)		311 (20.7)	206 (13.7)	183 (12.2)	267 (17.8)	349 (23.2)	188 (12.5)	
Intermountain Healthcare	**1,668 (10.8)**	218 (13.1)	1,450 (86.9)		506 (30.3)	145 (8.7)	128 (7.7)	266 (15.9)	490 (29.4)	133 (8.0)	
KPNC	**5,489 (35.4)**	438 (8.0)	5,051 (92.0)		582 (10.6)	838 (15.3)	1,076 (19.6)	1,193 (21.7)	1,384 (25.2)	416 (7.6)	
KPNW	**1,028 (6.6)**	82 (8.0)	946 (92.0)		305 (29.7)	135 (13.1)	104 (10.1)	181 (17.6)	238 (23.2)	65 (6.3)	
University of Colorado	**906 (5.8)**	90 (9.9)	816 (90.1)		411 (45.4)	51 (5.6)	44 (4.9)	96 (10.6)	280 (30.9)	24 (2.6)	
**Age group, yrs**											
18–49	**2,925 (18.9)**	160 (5.5)	2,765 (94.5)	0.34	1,315 (45.0)	72 (2.5)	136 (4.6)	502 (17.2)	816 (27.9)	84 (2.9)	2.73
50–64	**2,985 (19.3)**	212 (7.1)	2,773 (92.9)		1,006 (33.7)	223 (7.5)	280 (9.4)	567 (19.0)	805 (27.0)	104 (3.5)	
65–74	**3,240 (20.9)**	300 (9.3)	2,940 (90.7)		717 (22.1)	385 (11.9)	391 (12.1)	526 (16.2)	1,007 (31.1)	214 (6.6)	
75–84	**3,615 (23.3)**	410 (11.3)	3,205 (88.7)		599 (16.6)	475 (13.1)	551 (15.2)	636 (17.6)	1,072 (29.7)	282 (7.8)	
≥85	**2,737 (17.7)**	370 (13.5)	2,367 (86.5)		455 (16.6)	406 (14.8)	422 (15.4)	405 (14.8)	849 (31.0)	200 (7.3)	
**Sex**											
Female	**8,393 (54.1)**	747 (8.9)	7,646 (91.1)	0.06	2,147 (25.6)	861 (10.3)	966 (11.5)	1,437 (17.1)	2,500 (29.8)	482 (5.7)	0.19
Male	**7,109 (45.9)**	705 (9.9)	6,404 (90.1)		1,945 (27.4)	700 (9.8)	814 (11.5)	1,199 (16.9)	2,049 (28.8)	402 (5.7)	
**Race and ethnicity**											
Black or African American, NH	**1,788 (11.5)**	116 (6.5)	1,672 (93.5)	0.2	634 (35.5)	138 (7.7)	171 (9.6)	247 (13.8)	544 (30.4)	54 (3.0)	1.17
Hispanic or Latino	**2,394 (15.4)**	178 (7.4)	2,216 (92.6)		696 (29.1)	211 (8.8)	248 (10.4)	488 (20.4)	682 (28.5)	69 (2.9)	
Other,** NH	**1,500 (9.7)**	117 (7.8)	1,383 (92.2)		279 (18.6)	197 (13.1)	239 (15.9)	301 (20.1)	379 (25.3)	105 (7.0)	
Unknown	**239 (1.5)**	21 (8.8)	218 (91.2)		111 (46.4)	13 (5.4)	24 (10.0)	29 (12.1)	56 (23.4)	6 (2.5)	
White, NH	**9,581 (61.8)**	1,020 (10.6)	8,561 (89.4)		2,372 (24.8)	1,002 (10.5)	1,098 (11.5)	1,571 (16.4)	2,888 (30.1)	650 (6.8)	
**Documented prior SARS-CoV-2 infection^††^**											
Yes	**2,444 (15.8)**	141 (5.8)	2,303 (94.2)	0.2	641 (26.2)	213 (8.7)	251 (10.3)	413 (16.9)	819 (33.5)	107 (4.4)	0.18
No	**13,058 (84.2)**	1,311 (10.0)	11,747 (90.0)		3,451 (26.4)	1,348 (10.3)	1,529 (11.7)	2,223 (17.0)	3,730 (28.6)	777 (6.0)	
**SARS-CoV-2 status**											
Positive test result (case-patient)	**1,452 (9.4)**	1,452 (100.0)	0 (—)	—	434 (29.9)	122 (8.4)	165 (11.4)	231 (15.9)	444 (30.6)	56 (3.9)	0.26
Negative test result (control patient)	**14,050 (90.6)**	0 (—)	14,050 (100.0)		3,658 (26.0)	1,439 (10.2)	1,615 (11.5)	2,405 (17.1)	4,105 (29.2)	828 (5.9)	
**No. of monovalent mRNA vaccine doses received**											
None	**4,092 (26.4)**	434 (10.6)	3,658 (89.4)	0.1	4,092 (100.0)	0 (—)	0 (—)	0 (—)	0 (—)	0 (—)	—
2	**3,401 (21.9)**	322 (9.5)	3,079 (90.5)		0 (—)	48 (1.4)	81 (2.4)	194 (5.7)	3,020 (88.8)	58 (1.7)	
3	**5,003 (32.3)**	442 (8.8)	4,561 (91.2)		0 (—)	213 (4.3)	520 (10.4)	2,442 (48.8)	1,529 (30.6)	299 (6.0)	
4	**3,006 (19.4)**	254 (8.4)	2,752 (91.6)		0 (—)	1,300 (43.2)	1,179 (39.2)	0 (—)	0 (—)	527 (17.5)	
**Most recent dose product manufacturer**											
Pfizer-BioNTech	**7,088 (45.7)**	622 (8.8)	6,466 (91.2)	0.09	0 (—)	986 (13.9)	1,101 (15.5)	1,439 (20.3)	2,878 (40.6)	684 (9.7)	—
Moderna	**4,322 (27.9)**	396 (9.2)	3,926 (90.8)		0 (—)	575 (13.3)	679 (15.7)	1,197 (27.7)	1,671 (38.7)	200 (4.6)	
None	**4,092 (26.4)**	434 (10.6)	3,658 (89.4)		4,092 (100.0)	0 (—)	0 (—)	0 (—)	0 (—)	0 (—)	
**Any chronic condition**											
Yes	**14,649 (94.5)**	1,410 (9.6)	13,239 (90.4)	0.14	3,748 (25.6)	1,536 (10.5)	1,743 (11.9)	2,454 (16.8)	4,322 (29.5)	846 (5.8)	0.84
No	**853 (5.5)**	42 (4.9)	811 (95.1)		344 (40.3)	25 (2.9)	37 (4.3)	182 (21.3)	227 (26.6)	38 (4.5)	
**≥1 chronic respiratory condition**											
Yes	**9,246 (59.6)**	921 (10.0)	8,325 (90.0)	0.09	2,324 (25.1)	1,032 (11.2)	1,150 (12.4)	1,527 (16.5)	2,673 (28.9)	540 (5.8)	0.43
No	**6,256 (40.4)**	531 (8.5)	5,725 (91.5)		1,768 (28.3)	529 (8.5)	630 (10.1)	1,109 (17.7)	1,876 (30.0)	344 (5.5)	
**≥1 chronic non-respiratory condition**											
Yes	**14,119 (91.1)**	1,369 (9.7)	12,750 (90.3)	0.13	3,530 (25.0)	1,514 (10.7)	1,707 (12.1)	2,385 (16.9)	4,157 (29.4)	826 (5.9)	1.07
No	**1,383 (8.9)**	83 (6.0)	1,300 (94.0)		562 (40.6)	47 (3.4)	73 (5.3)	251 (18.1)	392 (28.3)	58 (4.2)	
**ICU admission**											
Yes	**2,566 (16.6)**	182 (7.1)	2,384 (92.9)	0.13	751 (29.3)	229 (8.9)	298 (11.6)	447 (17.4)	727 (28.3)	114 (4.4)	0.27
No	**12,936 (83.4)**	1,270 (9.8)	11,666 (90.2)		3,341 (25.8)	1,332 (10.3)	1,482 (11.5)	2,189 (16.9)	3,822 (29.5)	770 (6.0)	
**Receipt of invasive mechanical ventilation**											
Yes	**1,579 (10.2)**	97 (6.1)	1,482 (93.9)	0.14	567 (35.9)	111 (7.0)	126 (8.0)	226 (14.3)	497 (31.5)	52 (3.3)	0.75
No	**13,923 (89.8)**	1,355 (9.7)	12,568 (90.3)		3,525 (25.3)	1,450 (10.4)	1,654 (11.9)	2,410 (17.3)	4,052 (29.1)	832 (6.0)	
**In-hospital death^§§^**											
Yes	**466 (3.0)**	51 (10.9)	415 (89.1)	0.03	129 (27.7)	61 (13.1)	57 (12.2)	56 (12.0)	138 (29.6)	25 (5.4)	0.11
No	**15,036 (97.0)**	1,401 (9.3)	13,635 (90.7)		3,963 (26.4)	1,500 (10.0)	1,723 (11.5)	2,580 (17.2)	4,411 (29.3)	859 (5.7)	

**Abbreviations:** BV = bivalent; ICU = intensive care unit; KPNC = Kaiser Permanente Northern California; KPCHR = Kaiser Permanente Center for Health Research; MV = monovalent; NH = non-Hispanic; SMD = standardized mean or proportion difference.

* Hospitalizations with a discharge code consistent with COVID-19–like illness were included. COVID-19–like illness diagnoses included acute respiratory illness, respiratory signs or symptoms or febrile signs or symptoms using diagnosis codes from the *International Classification of Diseases, Tenth Revision*. Clinician-ordered molecular assays (e.g., real-time reverse transcription–polymerase chain reaction) for SARS-CoV-2 occurring ≤14 days before to <72 hours after the encounter date were included.

^†^ California (Sep 13–Nov 18, 2022), Colorado (Sep 13–Nov 7, 2022), Minnesota and Wisconsin (Sep 13–Nov 18, 2022), New York (Sep 13–Nov 18, 2022), Oregon and Washington (Sep 13–Nov 14, 2022), Texas (Sep 13–Nov 13, 2022), and Utah (Sep 13–Nov 18, 2022).

^§^ Vaccination was defined as having received the last monovalent or bivalent dose within the specified range of months/days before the hospitalization encounter date, which was the date of respiratory specimen collection associated with the most recent positive or negative SARS-CoV-2 test result before the admission date or the admission date if testing only occurred after the admission.

^¶^ An absolute SMD >0.20 indicates a nonnegligible difference in variable distributions between hospitalizations for vaccinated versus unvaccinated patients or for patients with a positive SARS-Cov-2 test result versus patients with a negative SARS-CoV-2 test result. For mRNA COVID-19 vaccination status, a single SMD was calculated by averaging the absolute SMDs obtained from pairwise comparisons of each vaccinated category versus unvaccinated. Specifically, it was calculated as the average of the absolute value of the SMDs for 1) vaccinated with only monovalent doses, ≥11 months earlier versus unvaccinated, 2) vaccinated with only monovalent doses, 8–10 months earlier versus unvaccinated, 3) vaccinated with only monovalent doses 5–7 months earlier versus unvaccinated, 4) vaccinated with only monovalent doses 2–4 months earlier versus unvaccinated, and 5) vaccinated with bivalent booster ≥7 days earlier versus unvaccinated.

** Other race includes Asian, Hawaiian or other Pacific Islander, American Indian or Alaska Native, other not listed, and multiple races. Because of small numbers, these categories were combined.

^††^ Previous SARS-CoV-2 infection was defined as having a positive SARS-CoV-2 test result (molecular or antigen) documented in the electronic health record ≥15 days before the hospital admission date. This does not capture infections in which testing was not performed or testing was performed but not available in the electronic health record, e.g., at-home testing.

^§§^ In-hospital death was identified at each individual site and was defined as a death while hospitalized and ≤28 days after admission.

## Discussion

Analysis of data from the multistate VISION Network found that during September–November 2022, when the BA.5 and other Omicron sublineages were the predominant circulating SARS-CoV-2 variants in the United States, bivalent booster doses (after receipt of 2, 3, or 4 monovalent doses) were effective in preventing medically attended COVID-19 compared with no previous vaccination among immunocompetent adults and provided additional protection when compared with previous monovalent mRNA vaccine doses only. VE was similar against COVID-19–associated ED/UC encounters and hospitalizations, which might reflect changing severity of hospitalized cases over time (*5*). Additional studies are needed to evaluate VE against outcomes such as COVID-19–associated severe respiratory illness or death. The IVY Network, an adult inpatient VE network, recently found higher estimated VE in adults aged ≥65 years compared with estimates for those aged ≥18 years included in this analysis (*6*). This might reflect differences in population subgroups evaluated. Long-term durability of bivalent booster vaccination protection also could not be assessed because of the short period of observation since bivalent dose receipt. In a recent analysis from VISION, during BA.4/BA.5–predominant circulation, 3-dose monovalent VE against COVID-19–associated hospitalization was observed to wane from 68% at 7–119 days after vaccination to 36% at ≥120 days (*5*). This might explain why, among patients who had received 2, 3, or 4 monovalent vaccine doses only, a longer interval since the most recent dose was associated with more relative protection after receipt of the bivalent booster dose.

Bivalent COVID-19 booster vaccines were developed to improve protection against circulating Omicron sublineages because of immune escape potentially associated with these subvariants and waning of monovalent vaccine-conferred protection over time (*7*). Real-world data suggest that bivalent boosters provide a modest degree of protection against symptomatic infection among adults compared with receipt of 2, 3, or 4 doses of monovalent vaccines only (*8*). Results from this study also demonstrate protection against ED/UC encounters and hospitalization during a period when BA.5 and other Omicron sublineage viruses predominated in the United States. With co-circulation of multiple respiratory viruses, including SARS-CoV-2, influenza, and respiratory syncytial virus, vaccination against respiratory diseases for which vaccines are available is especially important to prevent illnesses resulting in health care encounters and to reduce strain on the health care system (*9*). Additional studies will be critical to evaluating the durability of added protection, especially with circulation of sublineages of the BA.4/BA.5 Omicron variants such as BQ.1 and BQ.1.1.

The findings in this study are subject to at least six limitations. First, previous SARS-CoV-2 infection was not accounted for in this analysis. A large proportion of the population has now experienced SARS-CoV-2 infection which decreases the risk of future medically attended COVID-19 illness and might affect observed VE due to background immunity (*10*). Second, although models adjusted for relevant confounders, residual confounding is possible, including by behavioral differences and use of COVID-19 treatments such as nirmatrelvir/ritonavir (Paxlovid). Third, sublineage-specific VE could not be estimated. Fourth, this analysis did not compare product-specific bivalent booster VE estimates. Fifth, relative VE was estimated using the interval since receipt of last monovalent dose; this study was not statistically powered to estimate whether relative VE differed by number of previous monovalent vaccine doses received. Finally, because these data are from nine states, the patients in this analysis might not be representative of the entire population of the United States. Further, this analysis included adults who received bivalent booster doses shortly after authorization who might not be fully representative of the vaccine-eligible population. For example, over one half of bivalent booster recipients had previously received 4 monovalent vaccine doses. Additional VE studies are needed as coverage of bivalent boosters increases. 

In this early study of immunocompetent adults, significant protection from a booster dose of bivalent mRNA COVID-19 vaccine (after receipt of 2, 3, or 4 monovalent doses) compared with no vaccination was found, as well as significant relative benefits of a bivalent booster dose when compared with previous receipt of monovalent doses only. These findings support efforts to improve coverage with bivalent vaccines, although optimal timing for receipt of bivalent vaccine booster doses needs to be established. All eligible persons should stay up to date with recommended COVID-19 vaccination, including receiving a bivalent booster dose. In addition, persons should consider taking other precautions to avoid respiratory illness this winter season, including masking in public indoor spaces, especially in areas where COVID-19 community levels are high, to protect themselves and others and reduce strain on the health care system during an ongoing surge in multiple respiratory viruses.

**Summary**

**What is already known about this topic?**

Bivalent mRNA COVID-19 booster doses containing an Omicron BA.4/BA.5 sublineage component were recommended on September 1, 2022. The effectiveness of these updated vaccines against COVID-19–associated medical encounters has not been established.

**What is added by this report?**

Bivalent booster doses provided additional protection against COVID-19–associated emergency department/urgent care encounters and hospitalizations in persons who previously received 2, 3, or 4 monovalent vaccine doses. Because of waning of monovalent vaccine-conferred immunity, relative effectiveness of bivalent vaccines was higher with increased time since the previous monovalent dose.

**What are the implications for public health practice?**

All persons should stay up to date with recommended COVID-19 vaccinations, including receiving a bivalent booster dose if eligible.

## Footnotes

* SARS-CoV-2 variant proportions are monitored by CDC, and available online. https://covid.cdc.gov/covid-data-tracker/#variant-proportions

† Medical events with a discharge code consistent with COVID-19–like illness were included. COVID-19–like illness diagnoses were obtained from *International Classification of Diseases, Tenth Revision* (ICD-10) discharge codes. The specific codes used were: COVID-19 pneumonia: J12.81 and J12.82; influenza pneumonia: J09.X1, J10.0, J10.00, J10.01, J10.08, J11.0, J11.00, and J11.08; other viral pneumonia: J12*; bacterial and other pneumonia: J13, J14, J15*, J16*, J17, and J18*; influenza disease: J09*, J10.1, J10.2, J10.8*, J11.1, J11.2, and J11.8*; acute respiratory distress syndrome: J80; chronic obstructive pulmonary disease with acute exacerbation: J44.1; asthma acute exacerbation: J45.21, J45.22, J45.31, J45.32, J45.41, J45.42, J45.51, J45.52, J45.901, and J45.902; respiratory failure: J96.0*, J96.2*, and R09.2; other acute lower respiratory tract infections: J20*, J21*, J22, J40, J44.0, J41*, J42, J43*, J47*, J85, J85.0, J85.2, J85.3, J85.1, and J86*; acute and chronic sinusitis: J01* and J32*; acute upper respiratory tract infections: J00*, J02*, J03*, J04*, J05*, and J06*; acute respiratory illness signs and symptoms: R04.2, R05, R05.1, R05.2, R05.4, R05.8, R05.9, R06.00, R06.02, R06.03, R06.1, R06.2, R06.8, R06.81, R06.82, R06.89, R07.1, R09.0*, R09.01, R09.02, R09.1, R09.2, R09.3, and R09.8*; acute febrile illness signs and symptoms: R50*, R50.81, R50.9, and R68.83; acute nonrespiratory illness signs and symptoms: R19.7, R43*, R43.9, R51*, R51.9, M79.1*, M79.10, M79.18, R65*, R53.81, R53.83, R57.9, R41.82, R40*, R40.0, R40.1, R53.1, R11*, R11.0, R11.1, R11.10, R11.11, R11.15, R11.2, R21*, R10*, R10.0, R10.1*, R10.2, R10.3*, R10.8, R10.81, R10.81*, R10.84, and R10.9. All ICD-10 codes with * include all child codes under the specific parent code.

§
https://www.cdc.gov/vaccines/covid-19/clinical-considerations/interim-considerations-us.html

¶ Sites from the CDC-funded VISION Network that contributed data for this analysis were Baylor Scott & White Health (Texas), Columbia University Irving Medical Center (New York), HealthPartners (Minnesota and Wisconsin), Intermountain Healthcare (Utah), Kaiser Permanente Northern California (California), Kaiser Permanente Center for Health Research (Oregon and Washington), and University of Colorado (Colorado).

** The encounter date was either the date of collection of a respiratory specimen associated with the most recent positive or negative SARS-CoV-2 test result before the admission or visit date, or the date of the medical visit if testing occurred only after the admission or visit.

†† Encounters were excluded if a first mRNA booster dose (third dose) was received before it was recommended by CDC on September 23, 2021; the interval between the second and third doses was <5 months, a second mRNA booster dose (fourth dose) was received before it was authorized for adults aged ≥50 years on March 29, 2022; the interval between the third and fourth doses was <4 months; a bivalent booster dose was received before recommended and generally available to the public (September 6, 2022); or the interval between the last monovalent vaccine dose (second, third, or fourth dose) and the bivalent booster dose was <2 months. https://www.cdc.gov/vaccines/covid-19/clinical-considerations/interim-considerations-us.html

§§ Sixty–149 days was classified as 2–4 months, 150–239 days as 5–7 months, 240–329 days as 8–10 months, and ≥330 days as ≥11 months.

¶¶ 45 C.F.R. part 46.102(l)(2), 21 C.F.R. part 56; 42 U.S.C. Sect. 241(d); 5 U.S.C. Sect. 552a; 44 U.S.C. Sect. 3501 et seq.
